# Somatosensory integration in robot-assisted motor restoration post-stroke

**DOI:** 10.3389/fnagi.2024.1491678

**Published:** 2024-11-06

**Authors:** Legeng Lin, Wanyi Qing, Zijian Zheng, Waisang Poon, Song Guo, Shaomin Zhang, Xiaoling Hu

**Affiliations:** ^1^Department of Biomedical Engineering, The Hong Kong Polytechnic University, Kowloon, Hong Kong SAR, China; ^2^Research Institute for Smart Ageing (RISA), The Hong Kong Polytechnic University, Kowloon, Hong Kong SAR, China; ^3^Department of Applied Biology and Chemical Technology, The Hong Kong Polytechnic University, Kowloon, Hong Kong SAR, China; ^4^Department of Surgery, The Chinese University of Hong Kong, Shatin, Hong Kong SAR, China; ^5^Department of Computer Science and Engineering, The Hong Kong University of Science and Technology, Kowloon, Hong Kong SAR, China; ^6^Key Laboratory of Biomedical Engineering of Education Ministry, Zhejiang Provincial Key Laboratory of Cardio-Cerebral Vascular Detection Technology and Medicinal Effectiveness Appraisal, Department of Biomedical Engineering, School of Biomedical Engineering and Instrument Science, Zhejiang University, Hangzhou, China

**Keywords:** stroke, robot, rehabilitation, sensorimotor integration, somatosensory stimulation, movement recovery, neuroimaging, neuromodulation

## Abstract

Disruption of somatosensorimotor integration (SMI) after stroke is a significant obstacle to achieving precise motor restoration. Integrating somatosensory input into motor relearning to reconstruct SMI is critical during stroke rehabilitation. However, current robotic approaches focus primarily on precise control of repetitive movements and rarely effectively engage and modulate somatosensory responses, which impedes motor rehabilitation that relies on SMI. This article discusses how to effectively regulate somatosensory feedback from target muscles through peripheral and central neuromodulatory stimulations based on quantitatively measured somatosensory responses in real time during robot-assisted rehabilitation after stroke. Further development of standardized recording protocols and diagnostic databases of quantitative neuroimaging features in response to post-stroke somatosensory stimulations for real-time precise detection, and optimized combinations of peripheral somatosensory stimulations with robot assistance and central nervous neuromodulation are needed to enhance the recruitment of targeted ascending neuromuscular pathways in robot-assisted training, aiming to achieve precise muscle control and integrated somatosensorimotor functions, thereby improving long-term neurorehabilitation after stroke.

## 1 Introduction

Disruption of somatosensorimotor integration (SMI) after a stroke is a key barrier to motor restoration because SMI incorporates somatosensation (mainly tactile, proprioceptive, thermal, and painful perceptions) from the body and the external environment to shape movement in a closed-loop mode (Asan et al., 2021). The SMI coordination enables the execution of skilled tasks, learning of new skills, or relearning skills by neuroplasticity after neurological disorders, such as stroke (Papale and Hooks, 2018). It has been reported that the post-stroke SMI process exhibited weakened descending motor outputs to target muscles due to diverse compensatory neuroplasticity with disturbance of involuntary spasticity and overwhelmed ascending somatosensory feedback from a target muscle against those from the compensatory muscles, which could lead to abnormal movement patterns with muscular compensation and learned disuse in the long term (Hu et al., 2006; Zhou et al., 2021a).

In post-stroke rehabilitation, integrating somatosensory input into motor relearning is crucial for reconstructing SMI. Somatosensory in associated muscles and joints, primarily through tactile (such as massage, tapping to mechanoreceptors of the skin and muscles) and proprioceptive stimulations (such as joint positions in a motion and changes in muscle length) (Hartmann, 2009), could enhance the ascending neuromuscular pathways in the closed-loop SMI process, together with the descending motor outputs of the affected limb in repeated physical training (Asan et al., 2021). Moreover, precise SMI neuroplasticity for target muscles can reduce muscular compensation by minimizing learned disuse to achieve close-to-normal movement patterns in daily tasks.

Repetitive and goal-oriented physical practice is necessary for motor restoration post-stroke, even in the chronic phase. Thus, robot-assisted physical training has gained prominence in stroke rehabilitation because it offers precise and consistent delivery of highly repetitive movements when professional manpower is insufficient (Xing and Bai, 2020). However, current robots mainly emphasize the control precision of repetitive motor outputs. Seldom do robotic designs successfully recruit and/or control the somatosensory responses in the desired ascending neural pathways from the targeted, habitually disused muscles in post-stroke physical practices, which impedes the motor restoration requiring SMI compared to the interventions by human therapists who provide instructive pressing and tapping to muscles. It has been found that the post-stroke motor relearning process could be more efficient once somatosensory feedback was provided as tactile or proprioceptive cues in the practices (Handelzalts et al., 2021). Precise integration and reinforcement of muscular somatosensory pathways in robot-assisted rehabilitation requires quantitative measurement of somatosensory responses in real time to regulate the effective stimulation to a target muscle.

In this work, we discussed the technologies that may contribute to precise SMI in robot-assisted motor restoration poststroke from two different aspects, i.e., (1) the real-time quantitative measurement in post-stroke responses to sensory stimulations and (2) stimulation technologies for real-time control of somatosensory integration in robot-assisted training.

## 2 Quantitative somatosensory measurement in real time

Although somatosensation is perceived as subjective and context-dependent for individuals (ten Donkelaar et al., 2020), assessed by descriptive ordinal scales manually in clinical applications, neuroimaging techniques offer quantitative and objective measures to reveal the brain responses to external somatosensory inputs. Recent research achievements have demonstrated their capabilities in capturing dynamic patterns in response to external stimuli, which may be adopted in the SMI design of rehabilitation robots. Potential candidates include electroencephalography (EEG), functional near-infrared spectroscopy (fNIRS), and functional transcranial Doppler ultrasonography (fTCD). These technologies mainly reveal the brain dynamics by measuring the neuroelectrophysiological activities, i.e., EEG, and hemodynamics of the cerebrovascular system, i.e., fNIRS and fTCD. Although functional magnetic resonance imaging is currently a golden technology for brain imaging, it has not been included in this opinion as the current technology is hard to directly incorporate into robotic systems due to the restricted measurement environment and low temporal resolution for real-time applications.

### 2.1 EEG and fNIRS in assessing cortical responses to somatosensory stimulations

EEG and fNIRS emerge as potential candidates for real-time detection of the somatosensory responses in robot-assisted training because of their high temporal resolution in the measurement of brain responses, the low cost of the equipment, and the readiness of integration in robots (Li et al., 2022). Currently, they have not been used for SMI control in the robotic design because of the uncertainties on the dynamic signal patterns in response to different somatosensory stimulations after stroke.

Preliminary studies were conducted on analyzing EEG features in both time and frequency domains during somatosensory stimulations. For example, proportional relationships between the P300 amplitude of event-related potential (ERP) and stimulation intensities were observed in both focal vibratory stimulation (FVS) and neuromuscular electrical stimulation (NMES) to stroke survivors, as well as in unimpaired persons (Lin et al., 2024). In measuring proprioceptive responses, the N90 component of the somatosensory evoked potential during passive index finger movement is an effective marker, with its duration proportional to movement duration but unaffected by direction (Seiss et al., 2002). Although ERPs are the main EEG features for evaluating the cortical responses to somatosensory inputs, their accuracy requires high stimulation repetition for averaging to achieve a sufficient signal-to-noise ratio (Luck and Gaspelin, 2017), which limits further application in real-time processing. Compared with ERPs, EEG spectral features in the frequency domain have demonstrated greater potential for industrial applications due to calculation simplicity. For instance, studies have shown that EEG spectral powers in the theta (4 Hz−7 Hz) and beta (13Hz−30 Hz) bands could tell the fabric stimulations by cotton, wool, and nylon (Huang et al., 2020). Moreover, desynchronization in EEG mu wave (8 Hz−13 Hz) in the contralateral hemisphere showed a significant correlation with proprioceptive acuity in arm-reaching tasks assisted by a robot (Albanese et al., 2023). On the other hand, fNIRS measures characteristic changes in oxyhemoglobin (HbO) and deoxyhemoglobin in the cortical blood flow and reflects brain activities based on the neurovascular coupling mechanism (Zhang et al., 2024b). Temporary and local variations in the HbO concentration have been considered as a sensitive indicator of the cortical response to tactile stimulation. For example, Zhou et al. (2023) found higher HbO amplitudes in the dorsolateral prefrontal cortex during passive touch than active touch. Hong et al. (2017) successfully classified four different tactile stimulations based on HbO signal features (i.e., mean, peak value, and skewness).

Besides the respective features of EEG and fNIRS in response to somatosensory inputs mentioned above, there are also some mutual/similar analyses adopted in both EEG and fNIRS investigation, such as cortical lateralization for exploring the hemispherical asymmetry and functional connectivity (FC) analysis to understand the connectivities in the neurocircuitries in resting and dynamic states. For instance, fNIRS detected stronger hemispherical lateralization to the contralateral hemisphere after integrating vibrotactile stimulation into hand motor tasks in unimpaired subjects compared with those without vibrotactile stimulation (Du et al., 2022). Results of cortical lateralization were also obtained in EEG studies (Inanç et al., 2021). Moreover, studies have shown that FC differed between stroke and healthy individuals (Zhou et al., 2021b) and was associated with somatosensory deficits (Schlemm et al., 2023). Particularly, FCs of the supplementary motor area, the supramarginal gyrus, the primary somatosensory cortex, and the parietal opercular area have been found to be highly associated with proprioceptive function (Kenzie et al., 2024). Furthermore, dynamic FC, characterized by changes in both the strength and directionality of the connection between two cortical regions over rapid time scales (seconds to minutes) (Hutchison et al., 2013), showed significant sensitivity to tackle post-stroke alterations in somatosensory impairments in the subacute phase (Bruyn et al., 2023).

EEG and fNIRS, with their complementary spatial and temporal resolutions, allow for concurrent assessment of electrical and hemodynamic brain activity, making their combined use advantageous for comprehensively exploring the functional activity of the brain. In BCI designs, an EEG-fNIRS system showed improved classification accuracy on voluntary motor intentions (Yin et al., 2015). Studies on cortical responses to somatosensory stimuli also yielded multi-faceted information with the combined EEG-fNIRS features for further understanding of brain dynamics. For example, using EEG-fNIRS concurrent measurement, Chen et al. (2023) reported that HbO changes in the primary somatosensory cortex were mainly associated with tactile perceptions raised by different fractal surfaces. Meanwhile, the EEG entropy indicated a negative correlation with the comfortable extent when stimulated with the textures.

### 2.2 fTCD in somatosensory measurements

Compared to the measurement of cortical dynamics by EEG and fNIRS, fTCD provides continuous monitoring of cerebral blood flow velocity (CBFV) in the major cerebral arteries (D'Andrea et al., 2016). fTCD has been proposed for identifying real-time brain events based on the observations that the diameters of main-stem intracranial arteries remain almost unchanged (Ainslie and Hoiland, 2014), suggesting that CBFV changes are mainly related to the cortical activations supported by the blood supply via the neurovascular coupling function (Ball et al., 2024). Pioneering works have been carried out to explore fTCD features in somatosensory stimulations. For example, Hage et al. (2018) applied pneumotactile somatosensory stimulation to the right palm of unimpaired subjects. They found bilateral CBFV increases of about 20%, sharp decreases in pulsatility index of about 8%, and left lateralization of up to 3.9% in the stimulation. The magnitude of the initial increase in CBFV exhibited significant adaptation between subsequent trials.

### 2.3 Discussion on challenges in real-time somatosensory measurements

The aforementioned somatosensory measurements, based on the neuroimaging methods of EEG, fNIRS, and fTCD, have been primarily explored in unimpaired populations without systematic consolidation of their features for stroke rehabilitation. Furthermore, the feature extraction and recognition were performed manually in offline analysis, further hindering their integration into the real-time design of rehabilitation robots. Future studies are needed to address the following aspects: (1) standardizing somatosensory stimulation protocols specifically for post-stroke rehabilitation, (2) building up neuroimaging feature databases for the diagnostic classification of clinical impairments in the rehabilitative process, and (3) developing automatic signal processing techniques for real-time measurements. Somatosensory stimulations closely related to rehabilitative purposes should be further investigated in stroke subjects using standardized protocols. For example, FVS and NMES are common modalities in routine practice. It is essential to quantify their neurological responses to different dosages, durations, and stimulation patterns using neuroimaging methods for stimulation precision. Additionally, neuroimaging feature databases for stroke populations undergoing rehabilitation should be established. These databases will facilitate the development of machine-learning models for automatic feature recognition in real-time applications.

## 3 Somatosensory integration in robotic design

Based on the real-time quantitative measurement of the brain responses to somatosensory stimulations in post-stroke physical practice, the effective recruitment of the targeted SMI neurocircuitries could be monitored and regulated in robot-assisted training. In the current rehabilitation robots, the recruitment of the descending neural pathways is mainly implemented through two strategies in the robotic control design: (1) peripheral-effort-driven control, which promotes voluntary motor effort (VME) from the peripheral neuromuscular system, e.g., detecting electromyography (EMG) (Huo et al., 2023), to drive the robots, and (2) central-intention-driven control, by capturing desired brain activities in VME to control the robot, such as the BCI systems using EEG patterns in motor imagery (Khan et al., 2020). Although proprioception experiences, e.g., joint motions and positions, could be provided even in continuous passive movement by current robots, the neurological effectiveness of these afferent inputs has seldom been assessed and regulated in robot-assisted training. Moreover, effective control of somatosensory stimulation to paretic muscles for SMI neuroplasticity is still lacking in current robots, which could be implemented via direct stimulation to peripheral muscles and/or introduced by stimulations to the CNS through neuromodulation.

### 3.1 Somatosensory stimulation to the peripheral muscles and nerves

**Figure 1:**
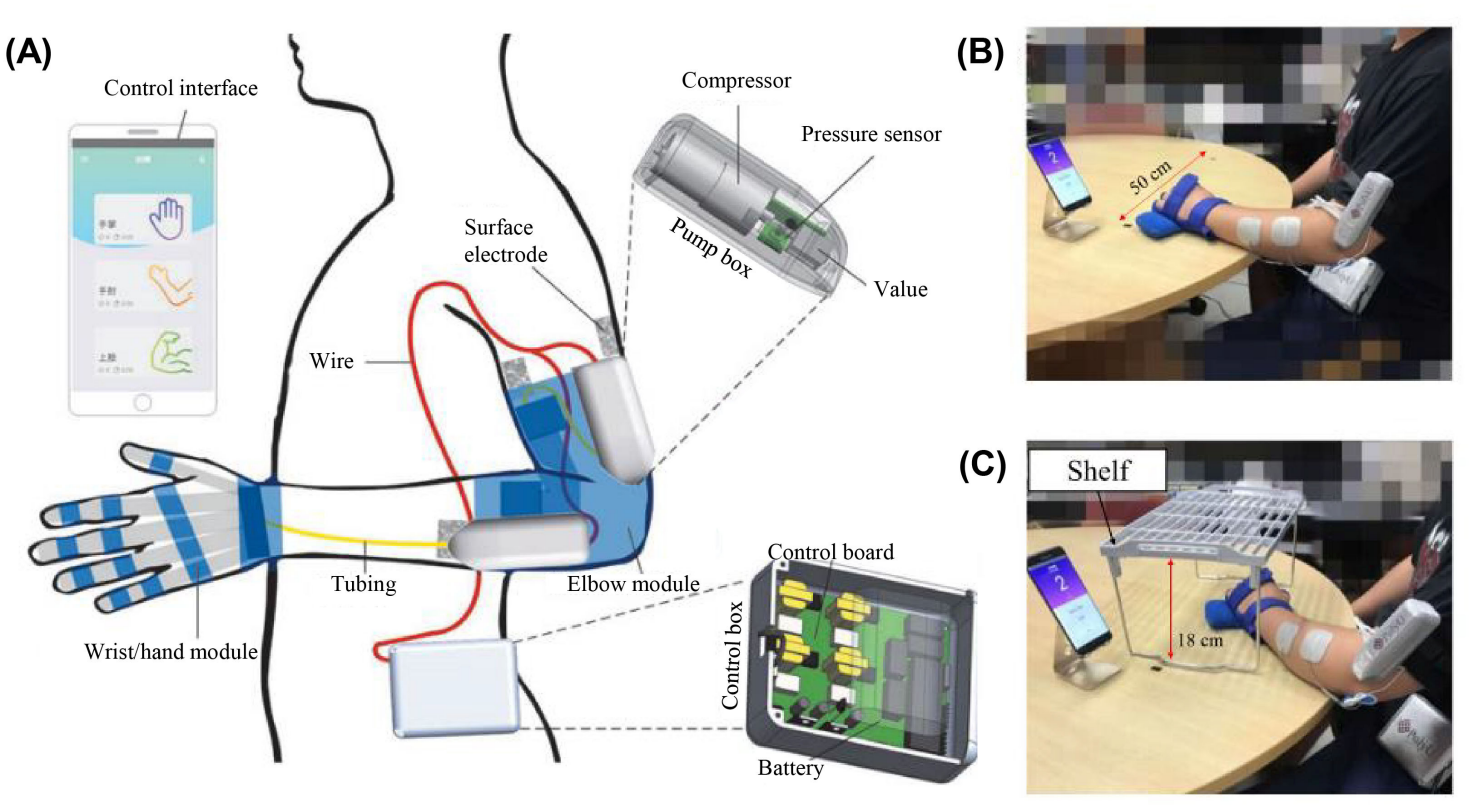
**(A)** Overview of the mobile exoneuromusculoskeleton for multi-joint upper limb telerehabilitation including wrist/hand and elbow modules (Nam et al., 2020). Home-based self-help training assisted by the exoneuromusculoskeleton with **(B)** horizontal and **(C)** vertical tasks using the wrist/hand module (Nam et al., 2021).

NMES, FVS, ultrasound stimulation, and infrared stimulation are promising modalities for muscle stimulation in robot-assisted training, mainly because of their ready-to-integrate platforms and non-invasive applications. Among them, NMES and FVS have been adopted for sensorimotor rehabilitation after stroke (Calabrò et al., 2017; Conforto et al., 2018). Preliminary NMES and robot hybrid systems (NMES-robot) indicated the benefits of introducing NMES to target muscles in addition to the mechanical assistance in limb motions, improving muscular coordination and leading to faster motor relearning compared to the traditional pure mechanical support (Qian et al., 2019). For example, Nam et al. (2020) designed a mobile exoneuromusculoskeleton for multi-joint upper limb telerehabilitation (Figure 1). The system integrated multi-channel NMES to upper limb muscles, together with pneumatic actuation to the elbow and fingers, to provide assistance in arm-reaching tasks. Residual EMGs in the paretic muscles were detected as VME to control the NMES and mechanical supports from the system. Patients with chronic stroke obtained significant motor gains in the upper limb after 20 sessions of self-help exoneuromusculoskeleton-assisted training in lab and home environments (Nam et al., 2020, 2021). However, the effects of somatosensory responses to NMES are ignored in the controls of NMES-robot currently. Compared to NMES's direct application for muscle contractions with a high stimulation intensity, transcutaneous electrical nerve stimulation (TENS) is mainly adopted for peripheral pain relief but also could enhance the somatosensory input by stimulating target peripheral nerves. For example, the REINFORCE system was designed to complement an exosuit's assistance by providing TENS on the medial tibial nerve and sural nerve on the feet to enhance somatosensation under the foot sole during the stance phase of walking (Basla et al., 2023). However, NMES and TENS as electrical stimulations widely excited neuroreceptors, e.g., nociceptors, which impedes the spatial precision and target specificity and the comfort in long-term usage.

FVS can also activate the post-stroke sensorimotor cortex by mainly depolarizing the mechanoreceptors in the skin and muscles (Lin et al., 2024), with less pain sensation compared to NMES. Similar to the NMES-robots, simple integration between FVS to target muscles and mechanical robots was proposed in the literature. For example, Calabrò et al. (2017) integrated on-and-off FVS to spastic upper limb muscles post-stroke together with robot-assisted limb movements; and the related clinical trial showed additional release of spasticity in the muscles. Different from NMES, whose motor effects could be easily inspected by the related muscle contractions, FVS mainly introduced sensations that have not been well evaluated in stroke survivors, as discussed previously. Future works are needed on the design of real-time regulation of the neurological effectiveness of each stimulating event in robotic control, based on the success in real-time measurement of the somatosensory responses in the CNS.

In addition to FVS and NMES, pulsed ultrasound stimulation (PUS) and pulsed infrared stimulation (PIRS) hold promise for modulating somatosensory processing in the peripheral nervous system. PUS produced both mechanical and thermal bio-effects and shared cortical-response characteristics similar to conventional somatosensory stimulus modalities, but with significantly improved spatial resolution and stimulation depth (Legon et al., 2012). This makes PUS an ideal stimulation candidate for the activation of small and/or deep muscles. On the other hand, PIRS has been explored for neurostimulation on the somatosensory cortex in animal models, e.g., rats, probably by its thermal bio-effects (Cayce et al., 2011). It showed promise as an alternative to electrical stimulation in peripheral applications with the advantages of contact-free or high spatial precision in stimulation. However, its peripheral neuromodulatory effects after stroke are not well understood. Besides the investigation of individual effects of the stimulation modalities, multi-modal somatosensory stimulation could be further explored for integration in SMI robotic design due to their complementary advantages, such as deep stimulation by PUS together with NMES and/or FVS.

### 3.2 Stimulation to the central nervous system

Apart from the direct stimulation to a target muscle, neuromodulatory stimulations to the CNS could elevate the efficiency in both the somatosensory and motor neural tracts, as well as their convergence in the cerebral cortex. Interventional stimulations, such as trans-spinal and transcranial stimulations by electricity, ultrasound, or magnetic field, are all potential modalities for SMI in robots.

Stimulations targeting SMI in the spinal cord, e.g., cervical spinal cord neuromodulation via trans-spinal electrical stimulation (tsES), could modulate the excitation of the intact spinal cord after stroke to facilitate the delivery efficiency of the residual neural drives from the ipsilesional hemisphere to a target distal muscle (Powell et al., 2023). For example, tsES enhanced the residual descending excitatory control, activated the local inhibitory circuits within the spinal cord, and reduced the cortical and proximal muscular compensation for stroke survivors (Zhang et al., 2024a). However, the post-stroke rehabilitative effects of tsES for the ascending pathway still remain unclear when targeting SMI for humans, even though it is effective in promoting local and cortical neuroplasticity changes through the activation of ascending corticospinal pathways (Marangolo et al., 2023). Further research is needed on the modulation of tsES on somatosensory feedback through afferent pathways, based on which the integration of tsES in robotic system design could then be further investigated in coordination with the peripheral somatosensory and movement interventions.

Transcranial neuromodulation techniques, including transcranial magnetic stimulation (TMS), transcranial direct current stimulation (tDCS), transcranial alternating current stimulation (tACS), and transcranial ultrasound stimulation (TUS), have been applied to promote neural plasticity and/or improve motor functions in stroke patients (Guo et al., 2022). Some of them have been adopted in timed paired stimulation (TPS) of sensory and motor systems for direct target sensorimotor integration, which arises from the basic learning properties of nervous systems (Asan et al., 2021). For example, TPS by TMS on the motor cortex paired with TENS on the peroneal nerve in the affected limb of participants with chronic stroke strengthened the evoked potentials from the cortex and improved gait patterns after the intervention (Uy et al., 2003). However, the direct paired stimulation of the motor and sensory cortex is seldom investigated targeting for SMI. Besides, in addition to their uncertain safety, the absorption and scattering of magnetic and electrical energy of TMS, tDCS, and tACS in the brain tissues limited the spatial resolution and penetration depth of the stimulations. Studies have found TUS a safer technique with deeper penetration and tinier spatial focus than those of magnetic and electric stimulations for modulation in the brain when applied to neurological and psychiatric disorders (Guo et al., 2022). Unfortunately, studies of TUS for stroke rehabilitation are sparse.

### 3.3 Discussion on challenges in somatosensory integration

Neuromodulatory stimulation to the CNS amplified the motor gain in physical training. For example, Asan et al. (2021) reviewed the catalyzing effects when central stimulations were paired with peripheral stimulations or traditional physical interventions for stroke survivors. However, these neuromodulatory methods have not been integrated with robot-assisted training. An optimized combination of CNS modulation, peripheral somatosensory stimulation, and robotic assistance may result in more effective SMI for motor restoration post-stroke, as synaptic efficiency could be elevated when stimulating neuromodulation concurrently applied during physical training (Cantone et al., 2021). More efforts are needed to understand the interactive mechanism between, or among, different stimulations with the baseline physical training assisted by a robot. Novel control strategies are also required to coordinate the different compartments in a real-time platform.

## 4 Conclusion

This article discussed the future directions of somatosensory integration in robot-assisted motor restoration after stroke in these two aspects: (1) Somatosensory measurement: real-time precise detection, standardized recording protocols, and diagnostic databases of quantitative neuroimaging features are required for real-time monitoring and regulation of the targeted somatosensory neurocircuitries in robots. (2) Somatosensory stimulation: regulated somatosensory stimulations on target muscles based on real-time somatosensory measurement and their optimized combination with robotic assistance and CNS neuromodulations are required in robotic design for enhancing the recruitment of targeted ascending neuromuscular pathways in robot-assisted training. In conclusion, it is time to implement somatosensory integration in robot-assisted motor restoration based on closed-loop SMI neuroplasticity to achieve precise muscular control and integrated somatosensorimotor functions for better long-term neurorehabilitation after stroke.

## Data Availability

The original contributions presented in the study are included in the article/supplementary material, further inquiries can be directed to the corresponding author.
